# Seeds of doubt: Mendel’s choice of *Hieracium* to study inheritance, a case of right plant, wrong trait

**DOI:** 10.1007/s00122-016-2788-x

**Published:** 2016-10-01

**Authors:** Ross Bicknell, Andrew Catanach, Melanie Hand, Anna Koltunow

**Affiliations:** 1 Plant and Food Research, Private Bag 4704, Christchurch, 8140 New Zealand; 2 Commonwealth Scientific and Industrial Research Organisation (CSIRO) Agriculture and Food, Private Bag 2, Glen Osmond, SA 5064 Australia

## Abstract

**Key message:**

**In this review, we explore Gregor Mendel’s hybridization experiments with** ***Hieracium*** **, update current knowledge on apomictic reproduction and describe approaches now being used to develop true-breeding hybrid crops.**

**Abstract:**

From our perspective, it is easy to conclude that Gregor Mendel’s work on pea was insightful, but his peers clearly did not regard it as being either very convincing or of much importance. One apparent criticism was that his findings only applied to pea. We know from a letter he wrote to Carl von Nägeli, a leading botanist, that he believed he needed to “verify, with other plants, the results obtained with *Pisum*”. For this purpose, Mendel adopted *Hieracium* subgenus *Pilosella*, a phenotypically diverse taxon under botanical study at the time. What Mendel could not have known, however, is that the majority of these plants are not sexual plants like pea, but instead are facultatively apomictic. In these forms, the majority of seed arises asexually, and such progeny are, therefore, clones of the maternal parent. Mendel obtained very few hybrids in his *Hieracium* crosses, yet we calculate that he probably emasculated in excess of 5000 *Hieracium* florets to even obtain the numbers he did. Despite that effort, he was perplexed by the results, and they ultimately led him to conclude that “the hybrids of *Hieracium* show a behaviour exactly opposite to those of *Pisum*”. Apomixis is now a topic of intense research interest, and in an ironic twist of history, *Hieracium* subgenus *Pilosella* has been developed as a molecular model to study this trait. In this paper, we explore further Mendel’s hybridization experiments with *Hieracium*, update current knowledge on apomictic reproduction and describe approaches now being used to develop true-breeding hybrid crops.

## Introduction

Gregor Mendel is justifiably referred to as the ‘Father of genetics’ due to his pioneering work on inheritance using the garden pea (*Pisum sativum*) as his model system. It is well known that his seminal paper describing this work was published in 1866, yet it received little attention until the work was ‘re-discovered’ by three separate researchers in 1900. What is less well-reported is that Mendel published a second paper on inheritance in 1869, describing his experiments with species of *Hieracium* subgenus *Pilosella* (Asteraceae). In that paper, he acknowledged that the principles of inheritance he described for pea could not be applied to the data he obtained using *Hieracium*. Fortunately, some of the letters written by Mendel to Carl von Nägeli, a prominent researcher of the time, survive and they clearly reflect the frustration Mendel felt over this outcome. We now understand that his principles of inheritance are correct and that they are applicable to all systems where sexual reproduction applies. Similarly, it is now understood that the patterns of inheritance Mendel observed in *Hieracium* were the outcome of facultative apomixis. It is now generally agreed that *Hieracium* species can be assigned into two *Hieracium* subgenera termed *Pilosella* and *Hieracium*. The plants used by Mendel were primarily of subgenus *Pilosella* which he referred to as “*Hieracium*” in his writings. In the interest of simplicity, when ‘*Hieracium*’ is used without qualification in this article, we are referring to ‘*Hieracium* subgenus *Pilosella’* Apomixis is now the focus of active genetic and molecular study in its own right, and *Hieracium* subgenus *Pilosella* is proving to be an excellent model to progress this work. In this review, we describe the work of Mendel on *Hieracium* and we reflect upon the contents of many of his letters to Nägeli. The advantages of *Hieracium* as a model are outlined and our current understanding of the genetics of apomixis is described. Finally, in respect to Mendel and to the profound contribution he made, we comment briefly on the circumstances of his life, the importance of science to him and the role of his work on *Hieracium* in particular.

## Mendel’s work with *Hieracium*

Although Gregor Mendel is primarily remembered for his hybridization experiments on garden pea (*Pisum sativum*) (Mendel 1869), there is evidence that he also hybridized species in the genera *Antirrhinum*, *Aquilegia*, *Carex*, *Cheiranthus*, *Cirsium*, *Geum*, *Hieracium*, *Ipomea*, *Linaria*, *Lychnis*, *Matthiola*, *Mirabilis*, *Phaseolus*, *Potentilla*, *Tropaeolum*, *Verbascum*, *Veronica*, *Viola* and *Zea* (Correns 1905; Mendel 1950). Also, he was interested in animal systems, particularly bees, in which he tried for many years to develop a strain that acquired more honey (Correns 1905; Mendel 1950). In his seminal paper on inheritance in *Pisum* (Mendel 1866), Mendel details his reasons for choosing this plant as his model system. He explains how a suitable system must be easy to emasculate and cross, be self-fertile yet not greatly influenced by “reduced fertility” (inbreeding depression), be easy to cultivate with a short generation time and be available in distinct forms that “are constant, and easily and certainly recognisable, and when their hybrids are mutually crossed they yield perfectly fertile progeny”. *Pisum* provided all of these advantages, and also it was available in a wide variety of true-breeding forms. Mendel obtained 34 lines and grew them for two years before selecting the seven he used for his subsequent experiments. Between the years 1856 and 1863, he conducted a series of inheritance studies requiring the cultivation of “more than 10,000 plants which were carefully examined”. The work culminated in the presentation of a lecture in 1865 and its publication in 1866.

From our perspective, Mendel’s work on inheritance was both insightful and comprehensive; however, from the perspective of his peers, it appears that it was regarded as being neither particularly convincing nor of much note. Mendel had 40 reprints made of his paper, which he posted to notable scientists of the day. Only Carl von Nägeli seems to have shown any real interest and he was only cautiously supportive (Correns 1905; Mendel 1950). The apparent indifference of Mendel’s peers is puzzling from a contemporary standpoint, especially as it came at a time when the nature of observed variation within and amongst species was being actively debated amongst European scientists. Darwin’s book discussing his theories on the origin of species (Darwin 1859), for instance, was published seven years prior and it attracted considerable attention.

Mendel had clearly hoped for a better reception to his findings. In a letter to Nägeli, he expressed his frustration in very measured terms, “I attempted to inspire some control experiments, and for that reason discussed the *Pisum* experiments at the meeting of the local society of naturalists. I encountered, as was to be expected, divided opinion; however, as far as I know, no one undertook to repeat the experiments”. Fortunately, many of the letters exchanged by Mendel and Nägeli survive and they provide a fascinating insight into Mendel’s efforts to get his work recognized (Correns 1905; Mendel 1950). One apparent criticism of his work was that it was unique to pea. In a letter to Nägeli dated 31 December 1866, Mendel wrote “I knew that the results I obtained were not easily compatible with our contemporary scientific knowledge, and that under the circumstances publication of one such isolated experiment was doubly dangerous; dangerous for the experimenter and for the cause he represented. Thus I made every effort to verify, with other plants, the results obtained with *Pisum*”. Mendel was being rather humble in this description of his efforts as his publication on pea also outlines his similar findings in bean (*Phaseolus*). It is clear, however, that he felt the need to provide further evidence to substantiate his claims and that he believed he needed a system unlike pea in which to do this. In light of the apparent care he took to select *Pisum* as a model for inheritance, it is intriguing that the systems he chose to verify his work were certainly not selected for their experimental tractability, but were more likely chosen based on the interests of his scientific peers.

The plants he chose to do this were *Cirsium*, *Geum* and *Hieracium*. Inheritance in *Geum* had been reported by the German botanist Carl von Gärtner in 1838 (Roberts 1919), but when Mendel tried to repeat his experiments in *Geum*, he was unable to replicate his results. *Cirsium* and *Hieracium* were favourite subjects of study amongst European botanists of the time. Nägeli, in particular, was a notable expert on their taxonomy, and he was especially interested in the role hybridization played in establishing patterns of speciation in these plants. There has been considerable speculation over whether Nägeli actively encouraged Mendel to study these plants or whether Mendel selected them before he contacted Nägeli to enlist his support. We cannot know for certain, but it is most likely that Mendel selected them before contacting Nägeli, hoping that his findings would be more favourably received if he could demonstrate them in well-accepted experimental models. It is, however, hard to imagine two plant groups less-suited to studying the underlying principles of inheritance than *Cirsium* and *Hieracium.* Both are daisies (Asteraceae) with minute flowers borne in terminal structures called capitula. Emasculation in daisies is very difficult as it requires dissection at the microscopic level. Mendel only had a simple lens to aid in his work, and his letters also indicate that he often worked under difficult lighting conditions. *Cirsium* is a genus of thistles, fiercely covered in needle-sharp spines. Also, the seedlings of *Circium* are delicate and easily lost. After considerable effort, Mendel finally decided that *Cirsium* was unsuitable and he chose to concentrate on *Hieracium* instead.

Other than the difficulties of handling such minute flowers, Mendel clearly believed that *Hieracium* was a suitable system for study. It was available in a wide variety of true-breeding forms, it was small, had a short generation time and it was easily handled (no gloves required). What Mendel could not have known, however, is that many *Hieracium* species are self-incompatible, polyploid and facultatively apomictic. Throughout the summers of 1866–1873, he laboured to establish *Hieracium* as an experimental system for studying inheritance. Over and over again, he collected the seeds of emasculated and crossed flowers only to observe that most of the progeny were maternal in type. We now know this is the result of asexual seed formation (apomixis), but Mendel, understandably, believed that it resulted from incomplete emasculation and self-pollination. Blaming himself for being inadequate, he sought to emasculate flowers at ever earlier stages, pushing the limits of his ability to see the structures he was dissecting. The technical difficulty of this task started to take its toll. In his letter of 15 April 1869 (Correns 1905), Mendel wrote to Nägeli noting “After having occupied myself a good deal during May and June with *H. auricula* and *H. praealtum*, a peculiar fatigue and exhaustion of the eyes appeared and reached a serious degree in spite of my immediately sparing my eyes as much as possible…Since then the affliction has luckily been almost completely lost, so that I am again able to read for long stretches at a time and can undertake the fertilization experiments with *Hieracium* as well as can be done without artificial illumination”. Later correspondence indicates that his eye condition persisted for many more years. We do not know how many emasculations Mendel performed on *Hieracium* as he only recorded the cases where successful hybridization was achieved. According to Correns (1905, 1924), he obtained hundreds of hybrids from 21 crossing combinations. In his brief publication detailing the work (Mendel 1869), he reports on the formation of ten hybrids from a total of six parental crosses.

We know today that *Hieracium* is a facultative apomict in which a small percentage of the progeny form sexually if the flower is pollinated at the right time. In the studied cases of *H. piloselloides* and *H. aurantiacum*, the levels of sexual seed formed following pollination were 2.0 and 2.4 %, respectively (Bicknell et al. 2003). Using this as a guide, it appears that Mendel must have emasculated in excess of 5000 florets to obtain the hybrids he used for further study. It was a monumental effort that took almost as long as his efforts with pea. Despite that effort, however, the results were clearly not what Mendel was hoping to see. When two apparently ‘true-breeding’ parents were crossed, he expected to see uniformity in their progeny, as observed when he crossed two inbred parental lines in pea. Instead, the sibling hybrids of *Hieracium* were highly variable. In his letter to Nägeli of 3 July 1870, he states “variants appeared in all those cases in which several hybrid specimens were obtained. I must admit to having been greatly surprised to observe that there could result diverse, even greatly different forms, from the influence of the pollen of one species upon the ovules of another species, especially since I had convinced myself, by growing the plants under observation, that the parental types, by self-fertilization, produce only constant progeny. In *Pisum* and other genera I had observed only uniform hybrids and therefore expected the same in *Hieracium.* I must admit to you, honoured friend, how greatly I was deceived in this respect”.

Of equal concern was the result he obtained when hybrid *Hieracium* plants were allowed to set seed following self-pollination. Mendel reasoned that the seed produced was the result of a self-fertilization event and he expected the seedlings would segregate in the manner seen in his F2 pea families. Instead, the progeny were typically highly uniform; “The second generation of the hybrids *H. praealtum (?)* + *H. aurantiacum and H. praealtum (Bauhini?)* + *H. aurantiacum* has flowered…Again the hybrids do not vary in these generations”. We now understand this to be an expression of apomixis. Collectively, his observations that, in *Hieracium*, true-breeding was not a reflection of homozygosity, and that F2 seedlings were not variable, led Mendel to conclude in July 1870 that: “On this occasion I cannot resist remarking how striking it is that the hybrids of *Hieracium* show a behaviour exactly opposite to those of *Pisum.* Evidently we are here dealing only with individual phenomena, which are the manifestation of a higher, more universal law”. In this statement, he appears to be admitting that the mechanisms are system-specific, although his comment about a higher law infers that he still held out hope that there was an explanation which would link his apparently disparate results from *Pisum* and *Hieracium*. Curiously, the idea of there being at least two types of inheritance persisted even after the rediscovery of Mendel’s work by de Vries (1900), Correns (1900) and von Tschermak (1900). In their acknowledgement of Mendel’s work, Bateson and Saunders (1902) continued to discuss the differences between the ‘*Pisum* type’ and the ‘*Hieracium* type’ of inheritance.

Mendel was an excellent administrator of his church, and in 1868, he was promoted to the prestigious position of Abbot of the Monastery of St Thomas in Brünn. Over the following years, this role demanded an increasing amount of his time to the expense of his work on hybridizing. In July 1870, he wrote “I have been the master on my own time for only a few days now, and am in a position to resume my favourite occupation, which I had to discontinue about the end of June last year, because of an eye ailment” (Correns 1905). In November 1873, it is clear that his commitments meant that he was largely unable to continue his scientific investigations; “I am really unhappy about having to neglect my plants and bees so completely. Since I have a little spare time at present, and since I do not know whether I shall have any next spring…” There is also evidence that his correspondence with Nägeli reduced significantly after this time, ending after two of Nägeli’s letters remained unanswered.

## Should Mendel have recognized that his *Hieracium* results could have been explained by parthenogenesis?

**Fig. 1:**
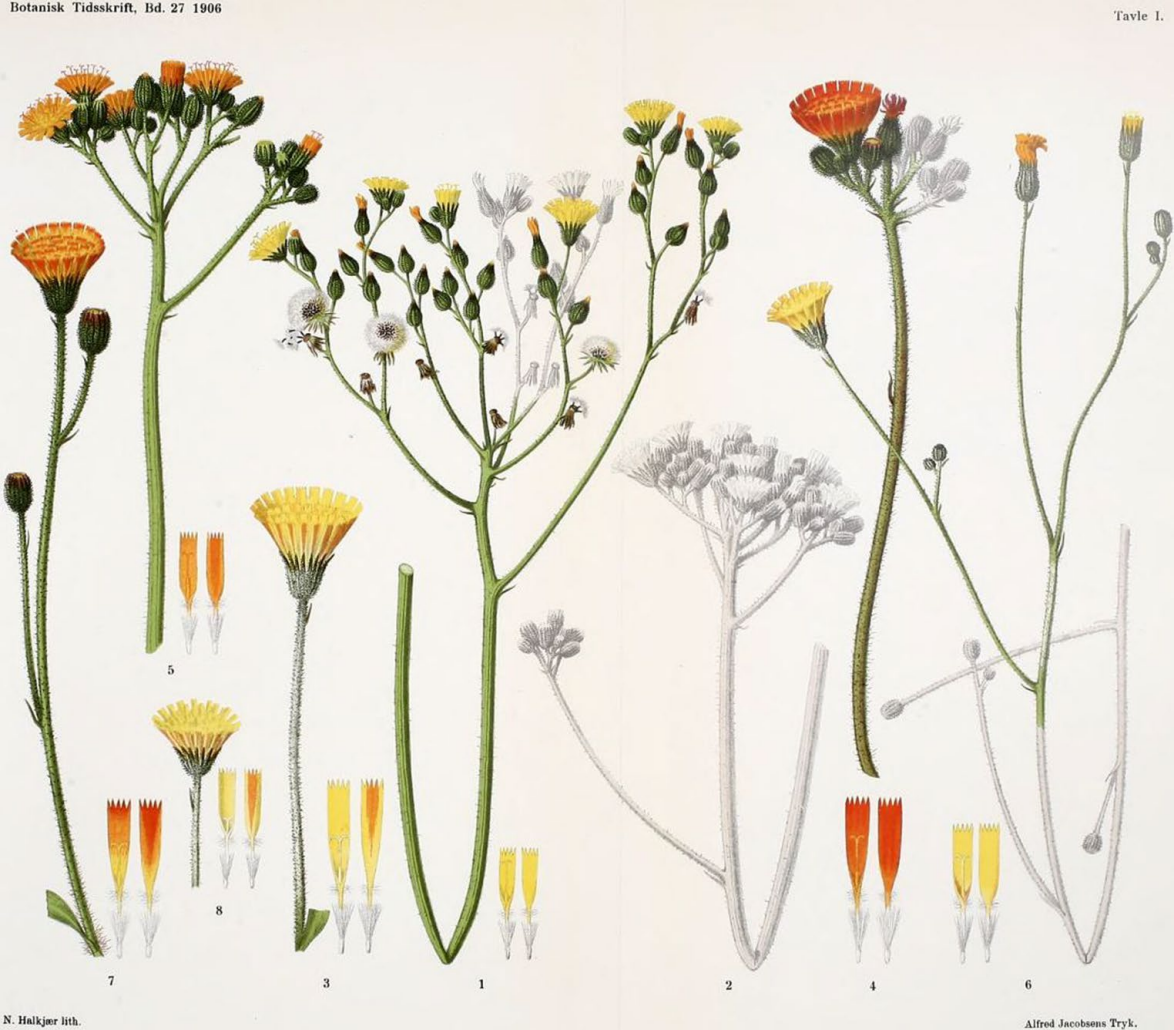
A figure reproduced from Ostenfeld and Rosenberg (1906) illustrating the parents used and the progeny derived from their hybridization experiments with *Hieracium*. 1 and 2: *Hieracium excellens*, 3: *H. pilosella*, 4; *H. aurantiacum*, 5: *H. excellens* × *aurantiacum*, 6: *H. excellens* × *pilosella*, 7: *H. pilosella* × *aurantiacum*, 8: *H. pilosella*. (Ostenfeld and Rosenberg (1906) Experimental and cytological studies in the Hieracia. I. Castration and hybridization experiments with some species of Hieracia. Botanisk Tidsskrift 27:225–248)

Parthenogenesis in flowering plants was actually first proposed prior to Mendel’s experimentation by Smith (1841) following his observations on the Australian native plant *Alchornea ilicifolia* (syn *Caelebgyne ilicifolia*). This species is dioecious. At the time, Kew Gardens held only a single female specimen which, despite the apparent lack of a pollen donor, formed abundant seed. Smith surmised that this species was parthenogenic, much like the well-known example of aphids. This record was, however, little more than a comment, and it was viewed with considerable scepticism from the scientific community of the time. It is not known if Mendel was familiar with examples of parthenogenesis in animals or the discussion regarding it in *Alchornea ilicifolia*, but in any case, an investigation into the reproductive modes of 12 potentially parthenogenic plants published 16 years later by Alexander Braun (1857) found no further examples. The prevailing view of the time, therefore, was that there was a “natural law of sexual reproduction” with parthenogenesis in *Alchornea ilicifolia* being a single exception. It was not until well after Mendel’s death that Murbeck (1897) and Juel (1898, 1900) determined the embryology of apomixis in the plants *Alchemilla* and *Antennaria alpina*, respectively, confirming Smith’s speculation and alerting researchers to the role asexual seed formation could have on determining patterns of natural variation. Finally, Ostenfeld and Rosenberg (1904, 1906, 1910) undertook a series of hybridization experiments with *Hieracium*, including repeats of Mendel’s crosses, and correctly attributed the patterns of progeny variation to a mixture of sexual hybridization and apomixis (see Fig. 1).

## Apomixis: the asexual formation of seeds

Apomixis is recorded in over 400 flowering plant taxa with representatives in at least 35 families (Carman 1997). Based on our knowledge of a small number of well-studied model apomictic systems, apomixis appears to be a modified form of sexual reproduction occurring in the ovule, which is the progenitor of the seed. During apomixis, embryos form from unreduced egg cells or somatic cells that have not undergone meiosis, and without fertilization. The seedling progeny are, therefore, essentially genotypic clones of the maternal parent. Most apomicts are facultative, so the ability to form seeds via a sexual route is retained to some degree in the plant. Ecologically, sexual and asexual sister species can co-exist, often with overlapping or adjacent ranges, and hybridization between the two can lead to the formation of new clonal lineages (apo-species). This occurs with *Hieracium* in central Europe, for instance, and is likely to be the reason why that region has such a diversity of forms, as observed by Nägeli and his peers.

The mechanisms employed to achieve clonal seed formation differ between plants and they have been grouped to sub-divide apomixis into a range of types (Crane 2001; Bicknell and Catanach 2015). Two main categories of mechanisms are recognized: sporophytic and gametophytic apomixis. In sporophytic apomicts, embryos arise directly from the somatic tissues of the ovule. Meiosis and fertilization are unnecessary for their initiation, and they rely on nutrients gained from fertilization in the adjacent sexually derived embryo sac to complete development. Citrus and mango typify this form of apomixis.

Gametophytic apomicts form an embryo sac mitotically without meiosis (apomeiosis). Within that embryo sac, an egg cell differentiates which then spontaneously divides, without fertilization, to give rise to an asexually derived embryo in a process termed parthenogenesis. Gametophytic apomicts are further sub-divided on the basis of the cell that initiates formation of the unreduced embryo sac. In ‘diplosporous’ types, the cell initiating the process is one that normally initiates the sexual program of meiosis, but instead initiates a program of mitotic embryo sac formation. This mechanism occurs in apomicts found in *Hieracium* subgenus *Hieracium*. In another mechanism of diplosporous embryo sac formation, the sexual progenitor cell initiates meiosis, but fails to complete it, undergoing a process termed meiotic restitution and then mitotic embryo sac formation. The common dandelion (*Taraxacum officinale*) is an example of this mechanism. Alternatively, in ‘aposporous’ types, a somatic cell termed an aposporous initial (AI) cell differentiates in the vicinity of cells undergoing meiosis and forms an embryo sac directly by mitosis. During this process, the nearby sexually developing embryo sac may or may not survive. In some aposporous apomicts, both apomeiotically derived embryo sacs and meiotically derived embryo sacs form, and seed can occasionally include embryos derived from both developmental pathways. However, *Hieracium* species in subgenus *Pilosella* are examples of aposporous apomicts where the sexual pathway is terminated prior to maturation during aposporous embryo sac formation.

An added consideration in the process of apomixis is that endosperm development is necessary for embryo development in flowering plants, which also includes apomictic species. Apomictic *Hieracium* in both subgenus *Hieracium* and *Pilosella* are rare examples of “autonomous apomicts” in which both embryo and endosperm development proceeds without fertilization. In most other apomicts, the endosperm will only form following the fusion of a male sperm cell with polar nuclei in the embryo sac, as occurs in sexual species. This process is termed “pseudogamy”. Seedlings arising from pseudogamy and autonomous seed formation are clonal, because there is no paternal contribution to the embryo. Apomictic grass species are typically pseudogamous. In pseudogamous *Pennisetum squamulatum* and *Pennisetum ciliaris*, one diploid polar nucleus is present in a characteristic four-nucleate, unreduced embryo sac. Fertilization then initiates endosperm formation, resulting in an endosperm with a 2 maternal:1 paternal genome ratio. The embryo possesses two maternal genome copies. Maintaining a 2 maternal:1 paternal genome ratio in the endosperm is critical for viable seed formation in many monocots. By contrast, in characterized aposporous *Hieracium* subgenus *Pilosella* species, fertilization-independent endosperm formation begins when the two unreduced polar nuclei fuse and the endosperm has a 4 maternal:0 paternal ratio indicating that a paternal genome is not essential for functional endosperm formation in these plants (Koltunow and Grossniklaus 2003; Hand and Koltunow 2014).

## *Hieracium* as a model apomict

There is an ironic post-script to the story of Mendel and *Hieracium*. The advent of molecular genetics led to renewed interest in the genetic basis of apomixis, and *Hieracium* is now an important model being used in this work (Koltunow et al. 2011; Hand and Koltunow 2014). In a curious reflection of history, *Hieracium* was chosen for this role partly because of its ease of cultivation, short stature, rapid generation time and the availability in a range of well-defined types (including both sexuals and apomicts). As these are characters he prized in this system we think, perhaps, that Mendel would have approved of this choice, despite the irony of now using this plant to study why allelic combinations sometimes do not segregate in accordance with his laws.

As described above, plants in the genus *Hieracium* (subgenus *Pilosella*) are either sexual or they are aposporous, gametophytic apomicts with autonomous seed formation. Both embryo and endosperm formation occur spontaneously in apomictic *Hieracium*; so, seeds form without pollination. They are also self-incompatible. Collectively, these features vexed Mendel’s efforts. In keeping with his observations in pea, he assumed that pollination had to be necessary; so, he interpreted seed production on emasculated plants as sign of failed technique. For the study of apomixis, however, it is an advantage since apomixis can be scored simply through the formation of progeny in the absence of pollination.

## The genetics of apomixis in *Hieracium*

As described above, *Hieracium* was extensively crossed by Gregor Mendel and he published his findings three years after his seminal paper on pea (Mendel 1869). He can, therefore, clearly be credited with being the first to study inheritance in this genus, even if he was not able to interpret the underlying cause of the inheritance patterns he observed. Following the pioneering work by Ostenfeld and Rosenberg (1904, 1906, 1910) recognizing the expression of apomixis in *Hieracium*, several studies have explored the genetic basis of apomixis in this genus. For viable, apomictically derived seed to form, both apomeiosis and parthenogenesis need to be functional and they need to operate successively within the ovule in conjunction with functional endosperm formation. Apomixis in *Hieracium* is controlled by two or three dominant loci. The genetic dominance of apomixis in *Hieracium* species was initially established using conventional experimental crosses, whereby apomictic *H. aurantiacum* and *H. piloselloides* were used as pollen parents in crosses with a sexual accession of *H. pilosella* which revealed dominant loci that independently and, respectively, co-segregated with apomeiosis and parthenogenesis (Bicknell et al. 2000). Although the methods used underestimated the role of apomeiosis in this study, it was determined that progeny fell into different classes based on the form of reproduction that they exhibited. Due to the independent actions of apomeiosis and parthenogenesis in *Hieracium*, at least four classes of progeny arise amongst the seed of a typical apomictic plant (Bicknell et al. 2003). There are the expected classes of sexual and apomictic progeny, but also intermediate classes exhibiting apomeiosis without parthenogenesis, or vice versa. Progeny from the two intermediate classes are viable but often weak and/or grossly different in their morphology to the sexually and apomictically derived progeny. In circumstances where apomeiosis occurs without parthenogenesis, the resulting unreduced embryo sacs need to be fertilized to activate embryogenesis. As a consequence, an embryo results carrying an additional genome. In the case of the reverse being true, whereby parthenogenesis occurs but apomeiosis does not, a reduced meiotic gametophyte may form and embryogenesis may then proceed parthenogenically. This results in a reduced plant, which has half the genomic complement of the parent.

In an effort to identify markers associated with apomeiosis and parthenogenesis, Catanach et al. (2006) undertook a deletion mutant screen in *H. praealtum*. Clonal seed was subjected to gamma irradiation and mutants were recovered that showed loss of apomeiosis, parthenogenesis, or both. After screening with AFLP, commonly lost markers were isolated, sequenced and used for the identification of BAC clones. The loci were termed *LOSS OF APOMEIOSIS* (*LOA*) and *LOSS OF PARTHENOGENESIS* (*LOP*).

Autonomous endosperm formation was seen to co-segregate extremely tightly with parthenogenesis at *LOP* in *H. praealtum*, and was generally assumed to be epistatic. Recently, Ogawa et al. (2013) conducted crosses between apomictic *H. piloselloides* and *H. aurantiacum* with sexual plants and also *H. praealtum* deletion mutant plants, identifying two recombinants exhibiting autonomous endosperm formation which had lost both apomeiosis and parthenogenesis. Backcrossing to a sexual species indicated that the trait of autonomous endosperm formation, denoted *AutE*, was a dominant locus. These analyses suggest that perhaps both processes of autonomous endosperm formation and parthenogenesis are in close linkage at *LOP* as identified in *H. praealtum*.

Analysis of apomixis in *Hieracium* subgenus *Pilosella* species collectively involving phenotypic characterization of segregants and mutants in conjunction with cell biological marker analyses and targeted inhibition of growth in ovule cell types shows that the initiation of sexual meiosis in ovules is required to activate the function of the *LOA* locus in somatic ovular cells. This may reflect signals from cells involved in meiosis itself, and/or signals from supporting sporophytic ovule cell types not involved in embryo sac elaboration (Koltunow et al. 2011). Importantly, deletion of apomixis loci results in a reversion to functional sexual reproduction, indicating that in *Hieracium* subgenus *Pilosella* apomicts, sexual reproduction is the default reproductive state and sexual reproduction is not functionally compromised. The identified apomixis loci enable the elaboration of a modified sexual pathway in a lineage initiated by the formation of an AI cell that avoids meiosis and fertilization and that suppresses sexual reproduction (Koltunow et al. 2011).

## The genetics of apomixis in other systems

In addition to *Hieracium*, a range of other natural apomictic species are being studied with the aim of isolating causal genes. In most systems, the components of apomixis are controlled by single, dominant loci, which makes the cloning of such loci and subsequent introduction into sexually reproducing crop species an appealing prospect. Although the mapping and characterisation of apomixis loci has been hindered by complications such as polyploid genomes, repetitive elements and a lack of recombination within the apomixis loci, some apomixis loci have been identified.

Besides the *LOA* locus of *Hieracium*, additional apospory loci have been identified in other species, although no causal function has yet been demonstrated. *Hypericum perforatum* (St. John’s Wort) reproduces via the apomictic components of apospory and parthenogenesis. In *Hypericum*, although apospory and parthenogenesis can be developmentally uncoupled (Barcaccia et al. 2006), the two components are genetically linked (Schallau et al. 2010). Apospory in *H. perforatum* is controlled by a dominant, simplex locus named *HAPPY* (for *Hypericum* APOSPORY), which was identified following AFLP-based analysis of a panel of ten apomictic and six sexual individuals (Schallau et al. 2010). Although a candidate gene denoted as ARIADNE, a potential RING-finger protein involved in ubiquitin-mediated protein degradation has been identified from the *HAPPY* locus, no causal role in apospory has yet been demonstrated.

A more complex inheritance model has been proposed for the aposporous grass species *Poa pratensis* (Kentucky bluegrass). Matzk et al. (2005) proposed a five-locus model of control in which the interaction of different elements either promoting or repressing apospory and parthenogenesis defined whether apomixis or sexuality was expressed. As with *Hypericum*, differential cDNA-AFLP analyses of apomictic and sexually reproducing *P. pratensis* were used to identify candidate apomixis genes (Albertini et al. 2004). *APOSTART* (*PpAPO1*) and *SERK* (*PpSERK*) are two genes believed to have a role in apospory in *P. pratensis*. The expression profile of *PpSERK* is consistent with the predicted spatial expression of genes involved in apospory, as it is expressed in cells neighboring the megaspore mother cell, which is the region where aposporous initial cells appear (Albertini et al. 2005).

Various approaches have also been taken to identify diplospory loci in natural apomicts that form diploid gametes through this mechanism. Classical map-based cloning has been employed in diplosporous *Taraxacum officinale* (common dandelion), where two unlinked, dominant loci have been shown to control diplospory and parthenogenesis (van Dijk et al. 2003). As in *Hieracium*, a third locus within *Taraxacum* is believed to control the apomixis component of fertilization-independent endosperm development (van Dijk et al. 2003). Map-based cloning is possible in *Taraxacum* due to the occurrence of recombination around the diplospory locus (*DIP*), and focused fine mapping efforts are being used to clone *DIP* (Vijverberg et al. 2010).

An evolutionary approach has been taken to identify the diplosporous locus within apomictic species of the genus *Boechera*, using the prediction that all diplosporous *Boechera* would share this locus, while the absence of such a locus would be expected within sexually reproducing *Boechera*. APOLLO is an exonuclease that was initially identified as a transcript differentially present between sexual and apomictic *Boechera* genotypes (Corral et al. 2013). Furthermore, analysis of 1649 *Boechera* accessions show that an apomixis *APOLLO* allele is highly conserved in apomicts, but mostly absent in sexually reproducing accessions (Mau et al. 2015). A candidate gene for the development of unreduced pollen grains has also been identified in *Boechera* species, and is named *UPGRADE2*. *UPGRADE2* was similarly identified by microarray-based comparative gene-expression analysis across sexual and apomictic *Boechera* (Mau et al. 2015). Future functional exploration of *APOLLO* and *UPGRADE2* in apomictic *Boechera* together with the introduction of these genes in other eudicots will reveal the functional role of these candidate genes for apomixis induction and seed formation.

In the aposporous grass *Pennisetum squamulatum*, a single hemizygous non-recombining chromosomal region known as the apospory-specific genomic region (ASGR) is responsible for both apospory and parthenogenesis (Akiyama et al. 2004). While no causal apospory genes have been identified within the ASGR, this genome region has yielded a parthenogenesis candidate gene, termed *BABYBOOM* (*BBM*)-like, which has become the first apomixis gene to be functionally demonstrated following transformation into a related sexual species (Conner et al. 2015). The BBM-like gene present in the *P. squamulatum* ASGR (*PsASGR*-*BBML*) is expressed in egg cells prior to fertilization, which would be expected of a gene capable of parthenogenesis. Sexual pearl millet transformed with *PsASGR*-*BBML* are able to produce haploid offspring via parthenogenesis at a rate of 35–36 % (Conner et al. 2015). *PsASGR*-*BBML*, therefore, has the potential to be transformed into other crop species, particularly grasses, enabling the engineering of the parthenogenesis component of apomixis into crops.

## Why study apomixis?

### Can apomixis be installed into crops?

The potential benefits of apomixis to plant breeding have long been recognized. Apomixis would allow the preservation of any genotype within a single generation, and it would remove the need for multiple cycles of inbreeding to achieve uniformity in seed-propagated crops (Bicknell and Catanach 2015). Many horticultural crops are already cloned commercially, such as potatoes and grafted fruit trees, and for these plants, the above advantages already apply. The initial intended targets of apomixis technology, therefore, are seed-propagated crops, including wheat, rice, cotton, maize, soy and most forestry species. For apomixis to be useful, it would need to be installed in a format that was highly penetrant. Also, it is preferable that the installed format be inducible to permit either sexual or asexual reproduction, as this would facilitate the efforts of plant breeders. Finally, it would be valuable, although not essential, to use a mechanism that utilized autonomous endosperm formation so that cross-pollination was not necessary.

Apomixis is uncommon amongst crops. It is recorded in citrus, mango and cassava, but these are vegetatively propagated; so, apomixis is of limited value in their cultivation. Apomixis is also relatively widespread amongst tropical forage grasses such as *Tripsacum dactyloides*, *Panicum maximum*, *Brachiaria decumbens* and *Poa pratensis*. Amongst seed-propagated, field-grown food crops, however, it appears to be limited to some varieties of onion (*Allium* sp.) (Kojima and Nagato 1997) and possibly some beets (*Beta*) (Cleij et al. 1976).

To date, two approaches have been taken to introduce apomixis into crops: introgression using related species and de-novo construction of the trait using transgenesis. Introgression is limited to cases where a suitable donor species is available and it only allows for the installation of the form of apomixis present in that donor. Although apomixis is not generally available in the primary gene pool of most crops, some do have apomictic relatives. Attempts to transfer the trait through wide crosses have been reported for maize [from *Tripsacum dactyloides*; (Leblanc et al. 1995; Grimanelli et al. 1998)], millet [from *Pennisetum squamulatum*; (Dujardin and Hanna 1983; Roche et al. 2001; Singh et al. 2007)], wheat [from *Elymus rectisetus*; (Peel et al. 1997)] and beet [from *Beta lomatogona*; (Cleij et al. 1976)]. Often, the expression of the trait diminishes with each subsequent back-cross, and the only way to retain it at a measurable level is to accept the retention of a significant amount of donor DNA. The breeding effort that has come closest to achieving the introgression of apomixis is pearl millet (*Pennisetum glaucum*). Following eight generations of backcrossing, an apomictic line was obtained that contains all the pearl millet chromosomes and only the single apomixis locus-carrying chromosome from *P. squamulatum* (Singh et al. 2010). That plant line, however, is not phenotypically typical of pearl millet and apomixis is unstable. In summary, the potential to introgress apomixis from wild relatives into crops is limited and has proven difficult to achieve.

An alternative to introgression is to directly introduce apomixis loci into crop species through genetic transformation. This has recently been achieved with the parthenogenesis *PsASGR*-*BBML* gene in pearl millet (Conner et al. 2015). This strategy obviously requires identification of apomixis-controlling genes in wild apomict species. Encouragingly, the results of Conner et al. (2015) suggest that a single gene is sufficient for expression of an apomixis component, despite the association of the *PsASGR*-*BBML* gene with extensive repetitive chromosomal regions. This finding is supported by work in apomictic *Hieracium*, where it has been demonstrated that the repetitive chromosomal structure associated with *LOA* is not necessary for apospory (Kotani et al. 2014). A potential advantage of such a transgenic approach is that the sexual pathway of the crop species need not be irreversibly altered. Given that sexual and apomixis pathways co-exist in natural apomicts, the introduction of an apomixis gene(s) into a sexually reproducing species may similarly superimpose apomixis pathways upon the crop’s sexual reproduction pathway. In this way, it may be possible to introduce apomixis genes in an inducible format. A potential disadvantage of inducing asexual reproduction in crops through the transgenic introduction of apomixis genes is the possibility of incomplete expressivity conferred by the apomixis genes due to their origin from an adapted species. Although the known examples of apomixis-controlling genes appear to act as single dominant loci, a number of modifying elements are likely to affect expressivity of the trait in natural apomicts. Indeed, the introduction of *PsASGR*-*BBML* in pearl millet resulted in incomplete penetrance of parthenogenesis (35–36 %) (Conner et al. 2015). A further consideration may be the type of apomixis to introduce into crop species, given that they likely differ based upon the complexity of the genetic control. As sporophytic apomixis only involves a single component (autonomous development of embryos) compared to multiple components of gametophytic apomixis, it may be simplest genetically to introduce sporophytic apomixis into crops. Although no genes responsible for sporophytic apomixis have yet been identified, a single genomic locus has been identified in *Citrus* which is currently being explored by map-based cloning (Nakano et al. 2012).

Another approach to install apomixis into crops would be to directly alter genes involved in sexual development within the crop species so that the re-engineered reproductive pathway mimics apomixis. Mutants of sexually reproducing species have been identified that display phenotypes that mimic apomixis, and previous reviews have discussed the range of genes responsible for this phenotype (e.g., Barcaccia and Albertini 2013). To mimic apomeiosis, female gametes must not have undergone recombination, which occurs during the first meiotic division, and they must be diploid (i.e., unreduced). An example of a mutant with a phenotype reminiscent of apomeiosis is the *Arabidopsis dyad* (*switch1*) mutant, which is able to produce unreduced, non-recombined gametes, albeit at a low frequency (0.24 %) (Ravi et al. 2008). An *Arabidopsis* line capable of the same phenotype is the triple-recessive, homozygous *osd1*/*rec8*/*spo11*-*1* mutant named *MiMe* for ‘mitosis instead of meiosis’ (d’Erfurth et al. 2009). This mutant combination produces gametes with a phenotypic outcome similar to mitotic diplospory, but with a resultant 100 % penetrance of unreduced, non-recombined gametes, and importantly, *MiMe* plants are fertile (d’Erfurth et al. 2009). The creation of *MiMe Arabidopsis* plants and the potential to recreate this genotype in a dominant manner in other species is a promising first step towards engineering apomeiosis in crops.

The parthenogenesis component of apomixis could potentially be mimicked in crop species by altering the genes responsible for the suppression of embryogenesis in the absence of fertilization. This has been achieved in the ‘Salmon’ wheat lines which exhibit up to 90 % parthenogenesis due to a translocation event between the short arms of wheat chromosome 1B and chromosome 1R of rye (Tsunewaki and Mukai 1990; Matzk 1996). From this translocation, wheat ‘Salmon’ lines have lost the *Suppressor of parthenogenesis* (*Spg*) and *Restorer of fertility* (*Rfv1*) loci, while gaining a *Parthenogenesis* (*Ptg*) locus from rye. The genes involved in this process, however, are yet to be identified. An *Arabidopsis* mutant in which an unfertilized egg cell is capable of embryogenesis is *multicopy suppressor of ira1* (*msi1*) (Guitton and Berger 2005). *MSI1* is a WD40 repeat protein and a member of the polycomb repressive complex 2 (PRC2), which is involved in chromatin remodelling. However, haploid embryos produced by *msi1* mutants are non-viable and embryogenesis arrests when the embryo structure consists of approximately 20 cells (Guitton and Berger 2005). No other loss-of-function mutants capable of permitting embryogenesis from an unfertilized egg cell have been identified.

In addition to the production of unreduced, non-recombined gametes and parthenogenesis, an apomictic system in a crop species would, ideally, also allow endosperm development in the absence of fertilization. Genes from *Arabidopsis* known to suppress fertilization-independent endosperm formation include additional PRC2 genes such as *MEDEA* (*MEA*), *FERTILIZATION INDEPENDENT SEED* (*FIS2*) and *FERTILIZATION INDEPENDENT ENDOSPERM* (*FIE*). Loss of function of any of these genes results in the initiation of endosperm development in the absence of fertilization; however, endosperm formation does not proceed to completion and the seeds are non-viable (Chaudhury et al. 1997; Luo et al. 1999; Ohad et al. 1999).

An alternate method to autonomous seed formation is genome elimination following fertilization, which can be induced by crossing with a haploid inducer line. Haploid inducers have long been used in maize breeding to generate homozygous doubled haploids. The maize haploid inducer lines Stock 6 (Coe 1959) and indeterminate gametophyte (Kermicle 1969) can produce up to 2 % haploid progeny when used as the male and female parent, respectively. Improvements in haploid induction efficiency have been achieved through genetic crosses and selection, with modern maize haploid inducer lines exhibiting up to 10 % of haploid progeny [reviewed in Prasanna et al. (2012)].

In *Arabidopsis*, a haploid-inducing line was generated from a centromere-specific histone (*CENH3*) null mutant complemented by a green fluorescent protein-tagged *CENH3* variant (Ravi and Chan 2010). When the haploid inducer is crossed to wild-type, a proportion of the progeny are haploid with the wild-type genotype, demonstrating genome elimination at some point after fertilization. The haploid inducer line can produce up to 45 % of haploid progeny when used as a maternal parent (Ravi and Chan 2010) although viable seeds are generated at a low frequency. Improvements to seed viability have come at a cost to the efficiency of genome elimination (Marimuthu et al. 2011). This approach has particular appeal as *CENH3* is a conserved protein across all plant species, meaning it can potentially be applied to generate haploid inducers in any plant species. This was recently demonstrated in maize and barley, although in barley, multiple copies of *CENH3* complicated the haploid induction (Karimi-Ashtiyani et al. 2015; Kelliher et al. 2016).

In *Arabidopsis*, the *CENH3*-impaired haploid inducer has been combined with the *MiMe* and *dyad* mutants to demonstrate engineering of clonal reproduction through seeds (Marimuthu et al. 2011). When *MiMe* plants were crossed to the haploid inducer, low numbers of viable seed were formed; however, 98 % of the diploid progeny had chromosomes only from the *MiMe* parent, and heterozygosity of this parent was also retained in the diploid progeny. This breakthrough proof-of-principle study highlights a potential strategy for engineering apomixis into normally sexually reproducing crop species. Conservation of the *MiMe* and CENH3 proteins throughout plant species implies that this approach could be made specific to each target crop species and does not rely on the crop species having a wild apomictic relative. The *MiMe* genome elimination strategy produces clonal progeny as occurs through apomixis, albeit low in number; however, crossing is still required at each generation to produce clonal diploid lines. Future strategies could focus on enabling the progeny to be self-reproducing so that diploid, clonal progeny are produced at each generation.

### The evolution of apomixis: when sex loses its appeal

While relatively rare, apomixis, has separately evolved many times in diverse, and often highly successful taxa. Its evolution is, therefore, of interest to evolutionary and population biologists, and to apomixis specialists alike. One question of interest, which applies to asexual reproduction in general, is why would sexual reproduction, a system that serves life so well that it is almost ubiquitous, be superseded repeatedly by asexual reproduction? Sex, while costly, is very widely utilized, which infers that the benefits of sex should outweigh the costs (Otto 2009). In the cases where asexual reproduction now predominates, however, the reverse argument presumably applies.

In addressing this question, it is important to realize, as noted above, that apomixis is seldom ever an exclusive state. Most apomicts are facultative, with sexuality occurring at low levels alongside apomixis, and *Hieracium* is no exception (Asker and Jerling 1992; Hand et al. 2015; Bicknell et al. 2003). Furthermore, many apomictic species produce colourful flowers that attract pollinators and/or they provide pollinator rewards. The apomicts of *Hieracium*, for example, produce large colourful flowers that reward insect visitors with pollen, yet the mechanism of apomixis employed by *Hieracium* completely negates the need for pollination. Considered together, the continued production of sexually derived progeny and the production of structures that encourage hybridization, it appears that there is a selective advantage to retaining both sexual and asexual reproduction at the level of the individual plant (Hojsgaard and Hörandl 2015). It is possible that a level of residual sexuality provides an advantage by allowing the purging of accumulated deleterious mutations. A comparison of transcriptome sequences from apomictic and sexual *Ranunculus* species suggests that the apomicts do not accumulate deleterious mutations, which may be attributed to the maintenance of residual sexuality within these plants (Pellino et al. 2013; Hojsgaard and Hörandl 2015).

Residual sexuality also occurs at the population level. In common with many apomictic taxa, apomictic clones of *Hieracium* frequently grow near to, or amongst inter-fertile sexual populations and gene flow occurs between them (Fehrer et al. 2007). Hybrids are most likely in cases where a sexual biotype is the seed parent, but apomicts can also hybridize and the resulting progeny can be either apomictic or sexual (Houliston and Chapman 2001; Chapman et al. 2003). Apomixis enables the efficient multiplication of elite genotypes, through the preservation of adaptive allele combinations and gene complexes, and this is believed to favour apomicts in colonizing new habitats. Hörandl and Hojsgaard (2012) proposed that apomixis and sexuality may act in concert to favour species survival. It is possible that following geographical expansion mediated mainly by apomixis, new sexual populations may become established by reverting back to obligate sexual reproduction. In this way, apomixis would facilitate both the diversity and distribution of the taxa in which it resides.

The benefits of asexual reproduction are even more compelling when the alternative benefits of sex are weak. A typical circumstance where this might be the case is reduced fertility upon interspecific hybridization, and apomixis has been suggested to exist primarily as an escape from such infertility. Support for this hypothesis is apparent in apomicts being almost always polyploid and usually heterozygous (Asker and Jerling 1992). Polyploidy may be the norm for alternative reasons, such as haploid gamete lethality of apomixis genes (Nogler 1984). However, an interesting view of the advent of apomixis has been postulated by Carman (1997, 2007). The hypothesis centres on apomixis being induced by epigenetic stabilisation of asynchronous expression of duplicated genes from each parent upon wide hybridization. The apomictic condition is stabilized further by apomixis itself, which represses meiotic recombination that might serve to disturb gene asynchronies.

Another intriguing question associated with the evolution of apomixis is how did so many gametophytic apomictic systems evolve? In the systems studied to date, gametophytic apomixis typically operates as a two-component system. Separate, unlinked, dominant genetic loci control the avoidance of meiosis (apomeiosis) and the avoidance of fertilization in embryo formation (parthenogenesis) (Tas and van Dijk 1999; Catanach et al. 2006; Schallau et al. 2010). Even in the case of the ASGR of *Pennisetum*, that determines both apomeiosis and parthenogenesis (Ozias-Akins et al. 2003), the two components can be separated by rare recombination events within the ASGR (Conner et al. 2013). When fully functional apomixis applies, the entire genome moves through successive seedling generations as a single linkage group, and natural selection acts at the level of the genotype, not at the level of the allele. Apomixis, therefore, preserves complex allelic combinations, but how could a multi-component system arise when the expression of either apomeiosis or parthenogenesis without the other would progressively increase or decrease genomic content, respectively? In natural populations, such genotypes are not expected to persist for more than a very limited number of generations (Van Dijk and Vijverberg 2005). Some advantages to such transitional plants can be envisioned. Apomeiosis, at low levels, without parthenogenesis, may confer some benefit to the evolution of species as it favours the formation of new polyploid products (Leitch and Leitch 2008). On the other hand, in situations where infertility is an issue, parthenogenesis at least ensures reproduction, albeit through the formation of plants with lower ploidy. Another possibility is that perhaps only a single, or very limited number of generations are involved, but that the number of progeny produced in that time is very large. Apomicts are almost invariably perennial and many have alternative forms of asexual reproduction (Richards 2003). *Hieracium* subgenus *Pilosella* is no exception; it is perennial and can spread very effectively via the growth of stolons. An individual genet of *Hieracium* may survive for many decades, come to cover a substantial area of land and produce hundreds of thousands of seeds every year (Sailer et al. 2014). Over the course of a single generation, therefore, millions of seeds can form. Any plant expressing either apomeiosis or parthenogenesis alone could, therefore, “bide its time” producing millions of seeds until a chance mutation or hybridization event completed the base requirements for apomixis, and selection at the level of the genotype, as described above, was established.

## Concluding comments

One hundred and fifty years ago, Mendel published his seminal paper on inheritance, outlining his discoveries using the model systems pea (*Pisum sativum*) and bean (*Phaseolus vulgaris*). As is well recorded, he deduced from those studies the fundamental principles of inheritance that guide us today and he effectively created the academic field of genetics.

As commented upon by Bernado (2016), ideas in science tend to passage through distinct phases of discovery, excitement, realization and, ultimately, once sufficient understanding is gained and the discovery remains valuable, a phase of utilization in a wider social context. Mendel’s laws of inheritance certainly took this course, but they did so over a timeframe far more protracted than the modern examples provided by Bernardo. In particular, the time from discovery to excitement was longer than four decades which sadly meant that Mendel died long before his findings elicited any real excitement amongst scientists. From the tone of his letters, it is certain that Mendel was well aware of the significance of his work and he was clearly excited by their potential. The indifference of his peers must, therefore, have been frustrating. Two notable features of Mendel’s personality, however, appear to have been humility and determination. Throughout his correspondence, he carefully avoids criticizing his peers for their indifference and lack of insight. Instead, he faced their silence as a challenge. His comments and actions indicate that he believed that his work on pea was overlooked primarily because it failed to provide sufficiently compelling evidence to justify his conclusions. In particular, it could be interpreted as being of possible value to pea breeders but of no greater consequence.

In response, he endeavoured to verify his results in one of the most enigmatic of plants imaginable: the genus *Hieracium*. His choice of this plant appears to have been influenced by the hope that his principles of inheritance would be more widely accepted if he could demonstrate their action in a system that was a popular focus of study at the time. Mendel only ever wrote two papers on genetics: the first on pea (Mendel 1866) and the second on *Hieracium* (Mendel 1869). In that second paper, he remarks that *Hieracium* “possesses such an extraordinary profusion of distinct forms that no other genus of plants can compare to it”. Later, he notes that “no other genus has so much been written or have so many and such fierce controversies arisen, without as yet coming to a definite conclusion. It is obvious that no general understanding can be arrived at, so long as the value and the significance of the intermediate and transitional forms is unknown”. We believe that it was his hope that he might resolve that debate by demonstrating how hybridization, progressing in accordance with his principles of inheritance, was the underlying cause of the variation seen in this group of plants. If he had been right, then perhaps he would have been more accepted by his scientific peers. He was, however, unable to resolve this puzzle and his unique understanding of inheritance was subsequently ignored for a further 44 years. We now know that the underlying cause of the patterns of variation seen in *Hieracium* is facultative apomixis. Apomixis is now a trait attracting significant interest amongst geneticists worldwide, and *Hieracium*, Mendel’s unlikely experimental choice, is proving to be an excellent model system for this work.

As a final note on the story of Mendel, it has been inferred that his life was tragically influenced by disappointment when his findings concerning inheritance were first ignored and later could not be substantiated using *Hieracium* as a model plant. In response, it is argued, he turned to a life of administrative duty (Iltis 1932). There is considerable evidence, however, to suggest that this was not the case (Gustafsson 1969). First, it is important to understand that Mendel was not only interested in inheritance. He published many more papers on meteorology than he did on genetics, for instance, and he kept daily records of the weather well after he stopped his crossing experiments. Second, he was employed by the church and this was always his primary responsibility. He excelled at administration, rising to the role of abbot at Brünn which was a significant achievement, particularly given his lowly birth. Finally, and perhaps most tellingly, it is recorded that he was afforded a lavish funeral, and that the streets were lined with the people of the city in respect for a man who was widely acknowledged for his contribution to the welfare of the community (Gustafsson 1969). In summary, we believe that Mendel was widely appreciated during his life for both his scientific endeavours and for his role as a leader of his Catholic faith. It just took a much longer time for the full extent of his brilliance to be appreciated.

### **Author contribution statement**

AK and RB formulated the structure of the review, AK, RB, AC and MH wrote the review.
